# What does your partner want? Using a gender equality lens to assess partner support and involvement in family planning in Uganda

**DOI:** 10.1371/journal.pgph.0003264

**Published:** 2024-05-29

**Authors:** Bolanle Olapeju, Anna Passaniti, Paul Odeke, Zoé Mistrale Hendrickson, Judith Nalukwago, Pallen Mugabe, Leonard Bufumbo, Musa Kimbowa, Fiona Amado, Emmanuel Kayongo, Mabel Naibere, Nanah Nanyonga, Glory Mkandawire, Richard Mugahi, Tabley Bakyaita, Richard Kabanda, J. Douglas Storey

**Affiliations:** 1 Department of Preventive Medicine and Biostatistics, Uniformed Services University of the Health Sciences, Bethesda, Maryland, United States of America; 2 Department of Health, Johns Hopkins Center for Communication Programs, Behavior and Society, Johns Hopkins Bloomberg School of Public Health, Baltimore, Maryland, United States of America; 3 Department of Behavioral and Community Health Sciences, University of Pittsburgh School of Public Health, Pittsburgh, Pennsylvania, United States of America; 4 Johns Hopkins Center for Communication Programs, Kampala, Uganda; 5 Reproductive Health and Infant Division, Ministry of Health of Uganda, Kampala, Uganda; 6 Department of Health Promotion, Education and Communication, Ministry of Health of Uganda, Kampala, Uganda; University of Ghana, GHANA

## Abstract

It is unclear if there are any differences in the ways men and women perceive partner support in the context of family planning. The USAID-funded Social and Behavior Change Activity (SBCA) in Uganda explored male versus female priorities in the decision-making considerations and preferred measures of partner support related to family planning. Data were from a cross -sectional nationally representative telephone survey of 1177 men and women aged 18–49 years old in sexual partnerships. Key measures included current family planning use (Are you or your partner currently doing anything to prevent or delay becoming pregnant?); family planning decision-making considerations (In your experience, which of the following are the three most important considerations as you make family planning decisions?); and preferred partner support (What level of involvement would you like to see from your partner in your family planning decisions?). Multivariable logistic regressions explored factors associated with decision-making priorities and preferred partner support, adjusting for sociodemographic confounders. Two-thirds (66%) of men and women wanted a high level of involvement from their partner, which was associated with higher odds of using family planning (aOR: 2.46, 95% CI: 1.87–3.24). Specific ways partners could be involved included accompanying them to health services (39%), permitting them to get family planning services (26%), and jointly discussing family planning options (23%). Of note, more women wanted their partner to accompany them (45%) than men (33%) while more men (29%) wanted to jointly discuss options than women (15%). Social and behavior change interventions should operationalize partner support differently for men and women. Study findings were used to implement a health campaign that explicitly encouraged partner dialogue and support across the various life stages; empowering women with knowledge and skills to have honest conversations with their partners about birth spacing and timing.

## Introduction

Uganda has a projected population of over 40 million residing in its Central, Eastern, Northern and Western Regions [1]. There is a backdrop of unmet need [2] and inequitable gender norms [3,4] related to family planning in Uganda according to FP2030 and Bureau of Statistics data. However, male partner support is associated with a threefold increase in a woman’s subsequent uptake of family planning [5]. Over three-quarters of Uganda women noted partner support for their current family planning use while up to a third of non-users noted lack of partner support.

The trends in partner support for family planning in Uganda are similar to other sub-Saharan Africa contexts [6–9] where partner support is often assessed through the women’s perception of their partner’s opinion [10] and is typically measured in a variety of ways. Some studies assess partner support as communication regarding family planning [11,12], involvement in decision making [6], and permission to use contraception [13]. Other studies measure partner support from a logistical lens and explore the provision of resources such as payment for the contraceptive method [14,15] or transportation to the clinic [14]. In Uganda, women have expressed a general discontent with partner involvement in their reproductive and maternal health, citing financial dependency, gender disparities, lack of autonomy, poor communication and prevailing social norms as barriers to receiving optimal help and support [15,16].

Women’s perspectives of their partner’s support may not be an accurate reflection of the male partner’s actual position as stereotyping of men’s opinion of family planning has been noted [10]. For example, while women might view partner support as being escorted to the clinic, some men view escorting their partner as being controlled by the woman or being labelled as weak [17]. Nevertheless, analysis of matched female and male partner’s perceptions suggests that while women are poorly aware of their partners actual opinion, their perceived partner approval was a strong predictor of modern contraceptive use [10].

Limited studies in Uganda have explored partner support for family planning from the male partner’s perspective or provided an in-depth understanding of male and female differences in how partner support is defined and operationalized. In Uganda, men’s fertility preferences, attitudes towards family planning, and prevailing norms in their community influenced their support and involvement in family planning [17]. Other factors influencing male partner support in sub-Saharan Africa include religion, level of education and socioeconomic status [14]. Qualitative studies suggest that men in Uganda preferred less involvement in family planning, citing excuses of work engagements, discomfort being seen escorting the woman to the clinic and feeling out of place while in the family planning clinic [17]. Understanding differences in male versus female preferences for partners support is crucial to improving male involvement in family planning resulting in subsequent uptake and continuity of contraception [18].

This study explores male and female differences in partner support preference in Uganda. The study aims to i) investigate preferred measures of partner support related to family planning among males versus females; and ii) assess male versus female priorities in family planning decision-making considerations. This understanding can inform the design of tailored interventions to improve contraceptive use at multiple levels including men, women, community as well as the facility. For example, behavior change communication focusing on men [12,19,20], couples [21] and community members [22] has been shown to improve spousal communication, joint decision making and subsequent family planning.

A theoretical underpinning for this study is the social support theory [23], which refers to resources that people have access to, based on their relationships and social ties. Within the context of family planning, women may receive support from their partners such as advice and information, emotional support, and services [24,25]. The exchange of such social support within couples can influence their family-planning decisions or actions, such as whether or not to use a contraceptive method [26]. Our research investigates how social support among couples can be better quantified and leveraged in the design of relevant family planning programs for Ugandan men, women, and couples.

## Methods

### Ethics statement

The ethical review and approval of the study were conducted by the Institutional Review Boards (IRB) from the Johns Hopkins Bloomberg School of Public Health (IRB No. 00013837) and the Makerere University Institute of Public Health Higher Degrees, Research and Ethics Committee (No. 864). Before participating in the telephone survey, all respondents provided informed verbal consent which was documented by the trained data collectors.

### Overview

This study was part of formative research activities implemented by the Social and Behavior Change Activity (SBCA) in Uganda, a 2020–2025 program that envisions a Uganda where individuals and communities are healthy, resilient, and supported by strong and adaptable systems and institutions to lead productive lives. The program provides social and behavior change (SBC)-related technical assistance to the Ministry of Health and other stakeholders to design and implement SBC initiatives that contribute towards a healthy nation. Specific outcomes include a reduction in maternal and child mortality, malaria prevalence, total fertility rate, new HIV infections, and tuberculosis prevalence, and improved nutrition outcomes. This study was part of formative research to identify individual and social cultural determinants affecting the uptake of key desired health behaviors and practices, including family planning. Results of this study and other formative research have since been used to inform the design and implementation of contextually relevant SBC interventions.

### Study design and participants

Study data were drawn from a cross-sectional nationally representative telephone survey of adults aged 18–49 years in Uganda in December 1–12, 2020. The telephone survey was conducted due to limitations in face-to-face data collection during the COVID-19 pandemic in the context of high telephone ownership (about 77%) in Uganda. Study inclusion criteria included the following: i) Aged 18–49 years old and resides in one of the four regions of Uganda: Central, Northern, Western, and Eastern; ii) Communicates effectively in English or the local language (Alur, Ateso, Japhadhola, Luganda, Lumasaba, Luo, Lusoga, Runyankore, or Runyoro); iii) Provides informed consent; and iv) Has access to a mobile phone either personally or through someone in the household.

### Sampling and sample size

The sampling procedure included a probability proportional to size (PPS) sampling of enumeration areas (primary sampling units), stratified by region, and based on projected data from the most recent 2014 national census. Next, study enumerators visited the enumeration areas and worked with community leaders to acquire a list of representative phone numbers for all households in the area. Specifically, study team members visited each household, briefly introduced the study, and requested the contact number of the head of the household or responsible adult, age and genders of household residents as well as the language spoken in the household. This was done to generate a validated sampling frame for the study and all procedures followed stipulated COVID-19 guidelines. The target number of telephone numbers was then randomly selected from the list of representative phone numbers within the enumeration area. After this, study data collectors systematically contacted, recruited, and interviewed sampled respondents. On the day of data collection, the interviewer would call and ask the household head for any eligible participant present and then introduce the study as needed before seeking informed consent. If a potential participant was not reachable, a replacement telephone number was then randomly selected from the list of representative phone numbers.

The telephone survey recruited a total of eight households each from all 175 enumeration areas for an overall sample of 1400 based on the following parameters: an outcome prevalence of 0.50 (for maximum variability); power = 0.80; alpha = 0.05; delta = 0.075; and a design effect of 1.5. This study explores family planning outcomes and thus excludes ineligible participants who are not in sexual partnerships (n = 233) for an analytical sample size of 1177 adults (53% men and 47% women). The unit of analysis is the individual and the data does not include couple dyads.

### Data collection

The telephone survey was administered in December 2020. Trained data collectors called participants, explained the purpose and benefits of the study, conducted eligibility screening, and obtained informed consent before proceeding with the survey questions. The survey interview lasted about 20–30 minutes and included questions on sociodemographic characteristics and behavioral outcomes related to family planning, malaria, maternal and child health, HIV, and COVID-19.

### Measures

Key variables included the following:

*Current family planning use* (yes versus no) was based on the survey question: Are you or your partner currently doing anything to prevent or delay becoming pregnant?

*Preferred partner support* was explored using two constructs: preferred level of partner support and specific partner support activities. The *preferred level of partner support* was assessed using the survey question: What level of involvement would you like to see from your partner in your family planning decisions? Response options included no, some, or high involvement.

*Specific partner support activities* were explored with the survey question: What specific involvement would you like to see from your partner in your family planning decisions? Response options included giving permission to go to the health center to get more information about family planning; accompanying me to the health center; discussing with me family planning options to consider; giving me permission to use family planning; paying for the family planning service.

*Family planning decision-making considerations* were assessed using the survey question: In your experience, which of the following are the three most important considerations as you make family planning decisions? Response options included discussing with my partner; choosing the right method; knowing a place where I can get family planning services; getting money to pay for family planning services; my partner or I do not approve of family planning; and other (specify).

Other variables included reasons for non-use of family planning (Why are you or your partner NOT doing anything to prevent or delay becoming pregnant?); sociodemographic characteristics by region (Central, Northern, Western, and Eastern); age in years (18–24, 25–39, and 40–49); location (urban versus rural); education (<primary, primary, ≥ secondary); and parity (0, 1–4, ≥5).

### Analysis

Cross-tabulations and tests of associations explored male and female differences in sociodemographic characteristics and family planning outcomes, decision-making priorities, and preferred partner support. Multivariable logistic regressions explored factors associated with decision-making priorities and preferred partner support. Covariates included current use of family planning, age, sex, region, rural versus urban residence, education, and parity.

## Results

### Description of study population

The study population presented in Table 1 comprised 53% men and 47% women. On average, the population was aged 25 to 39 years old (61%), lived in rural areas (72%), had less than secondary education (42%), and had one to four children (63%). There were significant sex differences in age, education, and parity. Specifically, women were more likely to be 25 to 39 years old or have more children while men were more likely to be educated.

**Table 1:** Description of study population (N = 1177).

	Male (N = 625)		Female (N = 552)		Total (N = 1177)		P- value
	No.	%	No.	%	No.	%	
**Region**							0.746
Central	160	25.6	139	25.2	299	25.4	
Eastern	166	26.6	157	28.4	323	27.4	
Northern	132	21.1	104	18.8	236	20.1	
Western	167	26.7	152	27.5	319	27.1	
**Age (years)**							0.011
18–24	55	8.8	66	12	121	10.3	
25–39	371	59.4	350	63.4	721	61.3	
40–49	199	31.8	136	24.6	335	28.5	
**Location**							0.825
Urban	178	28.5	154	27.9	332	28.2	
Rural	447	71.5	398	72.1	845	71.8	
**Education**							<0.001
Less than primary	152	24.3	210	38.0	362	30.8	
Primary	266	42.6	223	40.4	489	41.5	
Secondary or higher	207	33.1	119	21.6	326	27.7	
**Parity**							0.036
None	28	4.5	22	4.0	50	4.2	
One to four	375	60.0	371	67.2	746	63.4	
Five or more	222	35.5	159	28.8	381	32.4	
**Total**	**625**	**100.0**	**552**	**100.0**	**1177**	**100.0**	

### Family planning use and reasons for non-use

Table 2 presents rates of family planning use and reasons for non-use. Overall, about three- quarters (74%) of the study population (similar proportions among men and women) reported currently using family planning (defined as either them or their partner doing anything to prevent or delay becoming pregnant).

**Table 2:** Family planning use and reasons for non-use.

	**Male** **(N = 625)**		**Female (N = 552)**		**Total** **(N = 1177)**		**P-value**
**Currently using family planning**	No.	%	No.	%	No.	%	
Yes	463	74.1	405	73.4	868	73.7	0.782
No	162	25.9	147	26.6	309	26.3	
**Reasons for not using family planning among non-users**	**Male** **(N = 162)**		**Female (N = 147)**		**Total** **(N = 309)**		**P-value**
*Fear of side effects*	61	37.7	56	38.1	117	37.9	0.936
*Partner*, *family*, *or friends disapprove*	31	19.1	40	27.2	71	23	0.092
*Not knowing appropriate method to use*	15	9.3	18	12.2	33	10.7	0.396
*Forbidden by culture or religion*	15	9.3	13	8.8	28	9.1	0.899
*Family planning services are expensive/inaccessible*	11	6.8	9	6.1	20	6.5	0.812

Most common reasons noted by non-users of family planning (N = 309) included the fear of side effects (38%), disapproval from partner, family, or friends (23%), not knowing the appropriate method to use (11%), and cultural or religious concerns (9%), as well as the cost/inaccessibility of family planning services. None of these reasons differed significantly by sex.

### Preferred partner support and decision-making considerations for family planning

The contexts of partner support preferences and decision-making considerations for family planning are presented in Table 3. Equal proportions of men and women aged 18 to 49 years in Uganda (66%) preferred a high level of involvement from their partners. Specific ways that respondents noted their partners could be involved included accompanying them to health services (39%), permitting them to get family planning services (26%), and jointly discussing family planning options (23%). Notable gender differences included more women (45%) wanting their partner to accompany them than men (33%) and more men (29%) wanting to jointly discuss family planning options than women (15%).

**Table 3:** Preferred partner support and decision-making considerations for family planning (N = 1177).

	Male		Female		Total		P- value
**Preferred partner support**							
High involvement overall	405	64.8	367	66.5	772	65.6	0.544
Accompany to health center	209	33.4	247	44.7	456	38.7	<0.001
Joint discussion	183	29.3	85	15.4	268	22.8	<0.001
Permit to go to health center or use method	151	24.2	158	28.6	309	26.3	0.082
Pay for method	37	5.9	31	5.6	68	5.8	0.823
**Decision-making considerations**	No.	%	No.	%	No.	%	
Discussing with partner	525	84	479	86.8	1,004	85.3	0.180
Choosing the right method	459	73.4	415	75.2	874	74.3	0.495
Knowing where to get method	322	51.5	305	55.3	627	53.3	0.200
Getting money to pay for services	172	27.5	110	19.9	282	24	0.002

Most (85% each) men and women in Uganda aged 18 to 49 years equally considered discussing with their partners to be the biggest consideration for deciding to use family planning. Other important considerations equally noted by both men and women included choosing the right family planning method (74%), knowing where to get a method (53%), and getting money to pay for family planning services (24%). Interestingly, significantly more men (28%) compared to women (20%) viewed getting the money to get family planning services as an important consideration.

### Factors associated with preferred partner support in family planning

Table 4 presents characteristics of people with preferred levels and specific measures of partner support. Adults who preferred high levels of partner involvement (aOR: 2.46, 95% CI: 1.87–3.24) and those who preferred having their partner’s permission to go to the health center or use a method (aOR: 1.45; 95% CI: 1.06–1.99) were more likely to be current family planning users.

**Table 4:** Factors associated with preferred levels and specific activities of partner support (N = 1177).

	Overall Level of Support		Specific Activities of Support							
Characteristicsª	High involvement overall		Accompany to health center		Joint discussion		Permit to go to health center or use method		Pay for method	
	aOR	95% CI	aOR	95% CI	aOR	95% CI	aOR	95% CI	aOR	95% CI
Currently using family planning (reference: no)	2.46***	1.87–3.24	1.22	0.93–1.62	1.13	0.82–1.56	1.45*	1.06–1.99	0.81	0.45–1.44
Female (reference: male)	1.16	0.90–1.49	1.64***	1.28–2.09	0.46***	0.34–0.61	1.26	0.96–1.65	0.88	0.53–1.47
Eastern (reference: Central Region)	0.53***	0.37–0.76	0.95	0.66–1.36	0.98	0.65–1.46	1.52*	1.04–2.24	0.16***	0.07–0.39
Northern (reference: Central Region)	1.11	0.75–1.66	1.26	0.87–1.84	0.91	0.60–1.40	1.83**	1.23–2.74	0.10***	0.03–0.34
Western (reference: Central Region)	0.86	0.60–1.24	1.87***	1.33–2.64	0.80	0.53–1.19	0.81	0.54–1.21	0.65	0.36–1.17
25–39 years (reference: 18–24 years)	0.82	0.53–1.27	1.21	0.79–1.86	0.90	0.55–1.48	0.89	0.57–1.38	0.65	0.28–1.51
40–49 years (reference: 18–24 years)	1.03	0.63–1.67	1.29	0.80–2.06	1.05	0.61–1.81	0.75	0.46–1.24	0.48	0.19–1.23
Rural residence (reference: urban)	1.14	0.85–1.53	1.22	0.92–1.63	1.00	0.72–1.39	0.86	0.63–1.17	0.85	0.49–1.48
Primary (reference: less than primary)	0.93	0.69–1.25	1.05	0.79–1.41	0.85	0.60–1.21	1.08	0.78–1.48	1.27	0.68–2.36
Secondary or higher (reference: less than primary)	1.41	0.99–1.99	0.98	0.71–1.36	1.39	0.96–2.01	0.9	0.62–1.30	0.62	0.28–1.36
One to four children (reference: none)	1.11	0.59–2.09	1.15	0.61–2.19	1.49	0.68–3.25	0.72	0.37–1.39	1.26	0.35–4.53
Five or more children (reference: none)	1.12	0.58–2.17	1.02	0.52–1.99	1.56	0.69–3.51	0.83	0.41–1.65	1.45	0.38–5.55

ª Reference categories include male sex, Central region, 18–24 years of age, urban residence, less than primary education, nulliparity, and not currently using family planning.

*p≤0.05

**p≤0.01

***≤0.001.

Other important sociodemographic characteristics associated with preferred levels and specific measures of partner support included sex and Region. Females were more likely to want their partner to accompany them to the health center (aOR: 1.64; 95% CI: 1.28–2.09) but less likely to want joint discussion (aOR: 0.46; 95% CI: 0.34–0.61) compared to men. While regional differences in preferences were statistically significant, respondents’ age, education and parity were not statistically significant correlates of preferences.

### Factors associated with family planning decision-making considerations

Table 5 presents characteristics of people with different family planning decision-making considerations. Adults who viewed partner discussion (aOR: 5.11, 95% CI: 3.58–7.30), choosing a right method (aOR:3.38; 95% CI: 2.54–4.51), or knowing where to get a method planning (aOR: 1.98; 95% CI: 1.51–2.59) as important decision-making considerations were more likely to be current family planning users. Conversely, those who viewed partner approval as an important consideration for family planning were less likely to be family planning users (aOR: 0.30; 95% CI: 0.19–0.47).

**Table 5:** Factors associated with family planning decision-making considerations (N = 1177).

Characteristicsª	Discussion with partner		Choosing the right method		Knowing where to get method		Getting money to pay for services	
	aOR	95% CI	aOR	95% CI	aOR	95% CI	aOR	95% CI
Currently using family planning (reference: no)	5.11***	3.58–7.30	3.38***	2.54–4.51	1.98***	1.51–2.59	1.36	0.98–1.89
Female (reference: male)	1.37	0.96–1.95	1.16	0.88–1.54	1.2	0.94–1.53	0.65**	0.49–0.86
Eastern (reference: Central Region)	1.07	0.68–1.68	1.04	0.70–1.55	1.31	0.93–1.85	1.14	0.76–1.70
Northern (reference: Central Region)	5.25***	2.76–9.98	1.02	0.67–1.55	1.06	0.74–1.53	0.93	0.60–1.44
Western (reference: Central Region)	2.52***	1.52–4.18	1.48	0.98–2.21	1.18	0.84–1.66	1.33	0.90–1.96
25–39 years (reference: 18–24 years)	0.74	0.40–1.39	1.38	0.87–2.18	0.7	0.46–1.07	1.12	0.69–1.82
40–49 years (reference: 18–24 years)	0.75	0.38–1.47	1.17	0.71–1.94	0.76	0.48–1.21	0.71	0.41–1.23
Rural residence (reference: urban)	1.03	0.69–1.55	1.19	0.86–1.63	0.75*	0.57–0.99	0.96	0.69–1.32
Primary (reference: less than primary)	1.28	0.84–1.95	1.38	1.00–1.92	1.09	0.82–1.45	1.27	0.91–1.78
Secondary or higher (reference: less than primary)	1.27	0.79–2.04	1.56*	1.07–2.28	1.40*	1.01–1.93	1.11	0.76–1.63
One to four children (reference: none)	1.37	0.65–2.91	1.2	0.62–2.32	2.66**	1.40–5.09	2.22	0.91–5.42
Five or more children (reference: none)	1.34	0.60–3.00	1.12	0.56–2.24	2.40*	1.22–4.71	2.53*	1.01–6.36

ª Reference categories include (respectively) male sex, Central region, 18–24 years of age, urban residence, less than primary education, nulliparity, and not currently using family planning.

*p≤0.05

**p≤0.01

***≤0.001.

Other important sociodemographic characteristics associated with different family planning decision-making considerations included sex, region, education and parity. Of note respondents’ age and residence were not statistically significant correlates.

## Discussion

This novel study sought to address a key gap in the understanding of male and female perspectives related to partner support for family planning. Key findings included similar trends among men and women related to family planning use, reasons for non-use, decision-making priorities and preferred level of partner involvement. Notable sex differences were seen in some specific ways that men and women operationalized partner support as more men preferred to jointly discuss with their partner while women preferred their partner to accompany them to health center. Our study findings corroborate the existing literature on the importance of partner support for contraceptive use [5,7,8,17,18].

Study findings improve the current understanding of partner support for family planning and offer insights on how social support can be leveraged in partnerships to improve health and well-being, particularly related to family planning. Understanding couple dynamics and preferences related to family planning in Uganda may inform the design of tailored gender-aware SBC interventions that improve male involvement and reproductive health outcomes [17].

The findings suggest the need for interventions that push the narrative of partner support for family planning in Uganda using approaches that are sensitive to existing gender-based power imbalances among couples while seeking to encourage equitable partner communication [22]. USAID SBCA used these insights to inform the newly designed national multimedia umbrella family health campaign named “Happiness.” The campaign was developed using human-centered design processes leveraging the views of community members, leaders, and health providers in the program design and implementation.

The “Happiness” campaign also infuses behavioral science and economic approaches, such as narrative storytelling and nudges like special invitations or personalized messages to male partners, respectively. Campaign messages explicitly encourage partner dialogue and communication across the various life stages: during courtship, pregnancy, childbirth, and rearing. In addition, the campaign aims to empower women with knowledge and skills to have honest conversations with their partners about when to have children and how many to have and share their preferences related to family planning.

By engaging community gatekeepers and influencers, the campaign is also addressing negative gender norms and positioning family planning as not just a woman’s responsibility but a couple’s responsibility, as well as highlighting the fact that women have a right to use family planning regardless of partner approval. Additional opportunities to improve partner support, male involvement in family planning and the participation of men in decision-making and may include mobilization through community leaders, highlighting the economic benefit of family planning, packaging of the family planning interventions focus beyond family planning alone, and providing small incentives [22].

The study findings suggest that appropriate policies that operationalize partner support are needed for increased family planning uptake. At the community level, further efforts are needed to explore how existing by-laws such as those enforcing male attendance for antenatal visits [27] can also accommodate joint decision-making and gender equity related to family planning. This may include communication strategies during provider interactions with individual and couple clients. Given study findings highlighting respondents’ considerations about where to get a family method as well as the cost of services, policies should explore how family planning services can remain accessible to all community members regardless of gender, location or financial status.

Noteworthy strengths of this research include the use of nationally representative quantitative data employing a person-centered gender equality approach to understand how study populations define what partner support means to men and women in Uganda. However, we acknowledge some limitations that future researchers should aim to address. These include the use of cross-sectional data, which does not permit the inference of causality, as well as the use of telephone interviews, which may be prone to social desirability bias and the inability to observe non-verbal cues. In addition, family planning use in this study is notably higher than the national modern contraceptive use rates. This may be due to the fact that the study does not specify modern family planning methods. Furthermore, the telephone survey might have recruited more educated participants who are likely to be contraceptive users.

Use of family planning services depends on men and women’s abilities as sexual partners to negotiate contraceptive use as well as navigate power dynamics in partnerships and personal relationships in order to ensure shared responsibility in the uptake of family planning [28]. The study notes that specific partner support activities were preferred by less than half of the respondents. This signifies that there might be other ways to operationalize partner support for men and women in the Ugandan context, providing opportunities for future qualitative research.

Additional research should also investigate power dynamics and gender equity within relationships and the degree to which women feel empowered to have conversations about family planning. Such research findings can also be used to empower women to have conversations about family planning. Future research should uncover specific subgroup differences in partner support for family planning, such as how perceived partner support differs among younger versus older couples, type of sexual relationship (married versus unmarried), family planning needs (spacing versus limiting) as well as other cultural or psychosocial drivers of the heterogeneity in perceived partner support in Uganda.

## Conclusion

This innovative study explored the specific ways men and women in Uganda perceive partner support and make decisions in the context of family planning. Notable sex differences were seen in some specific ways that men and women operationalized partner support. Study findings suggest the need to operationalize partner support differently for men and women. These insights were used to implement a social and behavior change program that positioned family planning as couple’s responsibility, highlighted the importance of partner support and empowered couples to have honest conversations about their preferences.

## Supporting information

S1 Checklist Inclusivity statement. (DOCX)

S1 Data (XLS)
